# A Radiomics Nomogram for Non-Invasive Prediction of Progression-Free Survival in Esophageal Squamous Cell Carcinoma

**DOI:** 10.3389/fncom.2022.885091

**Published:** 2022-05-16

**Authors:** Ting Yan, Lili Liu, Zhenpeng Yan, Meilan Peng, Qingyu Wang, Shan Zhang, Lu Wang, Xiaofei Zhuang, Huijuan Liu, Yanchun Ma, Bin Wang, Yongping Cui

**Affiliations:** ^1^Key Laboratory of Cellular Physiology of the Ministry of Education, Department of Pathology, Shanxi Medical University, Taiyuan, China; ^2^College of Information and Computer, Taiyuan University of Technology, Taiyuan, China; ^3^Department of Thoracic Surgery, Shanxi Cancer Hospital, Taiyuan, China

**Keywords:** esophageal squamous cell carcinoma, computed tomography, progression-free survival, radiomics, nomogram

## Abstract

To construct a prognostic model for preoperative prediction on computed tomography (CT) images of esophageal squamous cell carcinoma (ESCC), we created radiomics signature with high throughput radiomics features extracted from CT images of 272 patients (204 in training and 68 in validation cohort). Multivariable logistic regression was applied to build the radiomics signature and the predictive nomogram model, which was composed of radiomics signature, traditional TNM stage, and clinical features. A total of 21 radiomics features were selected from 954 to build a radiomics signature which was significantly associated with progression-free survival (*p* < 0.001). The area under the curve of performance was 0.878 (95% CI: 0.831–0.924) for the training cohort and 0.857 (95% CI: 0.767–0.947) for the validation cohort. The radscore of signatures' combination showed significant discrimination for survival status. Radiomics nomogram combined radscore with TNM staging and showed considerable improvement over TNM staging alone in the training cohort (C-index, 0.770 vs. 0.603; *p* < 0.05), and it is the same with clinical data (C-index, 0.792 vs. 0.680; *p* < 0.05), which were confirmed in the validation cohort. Decision curve analysis showed that the model would receive a benefit when the threshold probability was between 0 and 0.9. Collectively, multiparametric CT-based radiomics nomograms provided improved prognostic ability in ESCC.

## Introduction

Esophageal cancer (EC) remains the seventh most frequently occurring cancer and the sixth most prevalent cause of cancer deaths globally (Bray et al., 2018). An estimated 477,900 new cases and 375,000 annual deaths have been reported in China, and most of them were esophageal squamous cell carcinoma (ESCC) (Chen W. et al., 2016). Most patients with ESCC are diagnosed at an advanced stage due to the vague symptoms in the early stage and have a meager 5-year survival rate (<20%) (Pennathur et al., 2013; Chen et al., 2017). However, surgery is still the most sanative treatment, and the 5-year survival rate of resectable EC treated with surgery alone is only 34–36% (Omloo et al., 2007). Hence, effective means to preoperatively predict the prognosis of patients with ESCC is necessary.

Prognosis survival evaluation of EC mainly depends on traditional Tumor Node Metastasis (TNM) staging. However, the TNM system only considers anatomical features and neglects the intrinsic factors of the tumor, resulting in an inaccurate prognosis (Wang et al., 2011). Then scholars started to collect clinical data, such as age, gender, body mass index (BMI), and the quality of life (Tang et al., 2013; Ng et al., 2014; Zeng et al., 2015; Zhang et al., 2016). However, the performance is still weak, for they failed to reflect the internals of tumors. Furthermore, prognostic evaluation by multi-omics approaches is based on molecular features of a small portion of tumor tissue, which limits the understanding of the heterogeneous tumor.

Radiomics, as a non-invasive, quantitative, and low-cost approach, can objectively and comprehensively evaluate tumor heterogeneity by converting medical images into high-dimensional, mineable, and quantitative imaging features via high-throughput extraction of data-characterization algorithms (Aerts et al., 2014; Gillies et al., 2016). These features can reveal disease progression, providing valuable information for personalized therapy and decision-support (Chicklore et al., 2013; Cameron et al., 2016; Huynh et al., 2016; Jin and Kong, 2016; Kotrotsou et al., 2016; Parekh and Jacobs, 2016; Ginsburg et al., 2017; Lee et al., 2017; Marin et al., 2017; Scalco and Rizzo, 2017; Shafiq-Ul-Hassan et al., 2017). Previous studies have shown that the radiomics signature alone or merged with clinical parameters could enhance predictive accuracy in cancers (Huang Y. et al., 2016; Huang Y. Q. et al., 2016; Zhang et al., 2017). Recently, the most widely-used imaging modality in radiomics is computed tomography (CT), which is universally used for preoperative diagnostics of ESCC. Due to the poor contrast resolution, it is not easy to distinguish the different histologic layers of the esophageal wall. However, it is believed that a lot of digital information could be deeply excavated through radiomics approaches.

In the present study, we developed CT-based radiomics as a novel approach for individualized, pretreatment evaluation of progression-free survival (PFS) in ESCC patients (stage I-III). Additionally, we sought to reveal the association between radiomics and clinical information.

## Materials and Methods

### Patients and Clinical Characteristics

Shanxi Medical University Review Board approved this retrospective study. The entire cohort was obtained from the Institutional Picture Archiving and Communication System (PACS) at Shanxi Cancer Hospital from February 2016 to October 2018. The patients who had histologically confirmed ESCC (TNM stage: I-III) and underwent surgery after diagnosis, underwent pretreatment CT scans from neck to abdomen and signed informed consent. All methods were carried out following the relevant guidelines and regulations.

To determine the patients that could be included, we developed the following criteria: (1) pathologically confirmed ESCC; (2) underwent surgery for ESCC; (3) standard contrast-enhanced CT was performed preoperatively; and (4) complete clinical and follow-up information was available. We randomly divided the patients into training and validation cohorts by a ratio of about 3:1. We trained models in the training cohort and validated them in the validation cohort.

Clinical characteristics including age, gender, tumor location (upper, middle, lower), drinking history, smoking history, genetic alterations, and pathologic features including depth of invasion, TNM stage, and lymph node metastasis information were collected from patient records. These clinicopathologic characteristics are presented in Table 1.

**Table 1:** Patient and tumor characteristics in the training and validation cohorts.

	**Training** **(*N =* 204)**	**Validation** **(*N =* 68)**	***P*** **-value**
Gender			0.539
Male	146 (71.6%)	46 (67.6%)	
Female	58 (28.4%)	22 (32.4%)	
Age			0.398
Median (interquartile range)	60.22	60.44	
≤ 56	63 (30.9%)	19 (27.9%)	
56–66	92 (45.1%)	27 (39.7%)	
≥66	49 (24.0%)	22 (32.4%)	
Location			0.452
Up	10 (4.9%)	5 (7.4%)	
Mid	135 (66.2%)	48 (70.6%)	
Down	59 (28.9%)	15 (22.1%)	
Drinking			0.662
Yes	75 (36.8%)	23 (33.8%)	
No	129 (63.2%)	45 (66.2%)	
Smoking			0.569
Yes	118 (57.8%)	42 (61.8%)	
No	86 (42.2%)	26 (38.2%)	
Genetic history			0.880
Yes	64 (31.4%)	22 (32.4%)	
No	140 (68.6%)	46 (67.6%)	
Invasion degree			0.887
Full layer	121 (59.3%)	41 (60.3%)	
Non-full layer	83 (40.7%)	27 (39.7%)	
TNM			0.556
I	19 (9.3%)	9 (13.2%)	
II	105 (51.5%)	36 (52.9%)	
III	80 (39.2%)	23 (33.8%)	
Lymph node metastasis			0.255
Yes	88 (43.1%)	24 (35.3%)	
No	116 (56.9%)	44 (64.7%)	

### Follow Up and Clinical Endpoint

All patients were followed up every 1–3 months during the first 2 years, every 6 months in years 2–5, and annually after that. To provide an efficient tool, which would allow earlier personalized treatment, we chose PFS as the endpoint (Sargent et al., 2005). We defined PFS from the first day of treatment to the date of disease progression (locoregional recurrences or distant metastases), death from any cause, or the date of the last follow-up visit (censored). The minimum follow-up time to ascertain the PFS was 2 months.

### CT Acquisition and Segmentation

All patients were underwent the contrast-enhanced CT using a 64-channel multi-detector CT scanner (LightSpeed VCT, GE Medical Systems, Milwaukee, Wis, USA). The acquisition parameters were: 120 kV; 160 mA; 0.5-s rotation time; detector collimation: 64 × 0.625 mm; field of view: 350 × 350 mm; and matrix: 512 × 512. After routine non-enhanced CT, contrast-enhanced CT was performed after a 25-s delay following intravenous administration of 85 mL of iodinated contrast material (Ultravist 370; Bayer Schering Pharma, Berlin, Germany) at a rate of 3.0 mL/s with a pump injector (Ulrich CT Plus 150, Ulrich Medical, Ulm, Germany). All images were reconstructed with a thick slice of 5.0 mm. We converted the image format from DICOM to NII for feature selection without any preprocessing.

Note that segmentation is required before the extraction of quantitative radiomics features; we performed three-dimensional manual segmentation using 3D-Slicer software (https://www.slicer.org/), an open platform for medical image processing. The chief physician of Shanxi Cancer Hospital, with more than 5 years' experience in interpreting chest radiology, outlined the tumor regions for each CT image layer. The tumor segmentation was guided and verified by the specialist. The region of interest (ROI) covered the whole tumor mass, was delineated on each CT slice, and used in subsequent feature extraction.

### Selection of Radiomics Features and Building of Radiomics Signature

We performed the calculation through our homemade Python scripts (Python3.6, https://www.python.org) for radiomics feature extraction based on the segmentation results. A total of 954 features were obtained by calling feature calculation in pyradiomics package (open-source python package; https://pyradiomics.readthedocs.io/en/latest/), which included the following fourcategories: (1) first-order statistics features; (2) size- and shape-based features; (3) texture features; and 4) wavelet features; and five typical matrixes: Gray-Level Co-occurrence Matrix (GLCM), Gray Level Run Length Matrix (GLRLM), Gray Level Size Zone Matrix (GLSZM), Gray Level Dependence Matrix (GLDM) and Neighboring Gray Tone Difference Matrix (NGTDM).

We built the radiomics signature with selected features in the training cohort. To reduce over-fitting or any types of bias, we applied the following two steps: First, the best features based on univariate statistical tests (2-sample *t*-test) between death and censoring groups in the primary cohort were selected and executed by using Matlab 2016b. Second, we used our homemade R scripts to select features that were most significant by using the least absolute shrinkage and selection operator (LASSO) method, which would be a suitable methodology for the feature selection through regression of high-dimensional data. Additionally, the accuracy of the prediction model could be improved by regularizing the features through penalized estimation. We added the L1 penalty term to the normal linear model, and the parameter lambda controls the complexity of regression. When the λ is large, it indicates no effect on the estimated regression parameters, while the λ gets smaller, most covariate coefficients were shrunk to zero. Then the remaining variables with non-zero coefficients were selected by the λ that the 10-fold cross-validation error was the most minor (Kumamaru et al., 2016; Vasquez et al., 2016). When performing 10-fold cross-validation, the training cohort was divided into 10 equal parts; each called a fold. Next, it will train a series of models. The first model was trained using the first fold as the test set and the other folds (2–10) as the training set. Then another model was constructed using 2nd fold as the test set and the1st, 3th-10th folds as the training set. The process is repeated with 3th-10th folds as a test set.

Finally, the radiomics signature was built by combining those variables in the primary cohort and validated in the validation cohort. The radiomics signature is a linear combination of selected features with respective weights, which would be calculated as a factor (Radiomics score, Rad-score) for the further prediction model. The Rad-score calculated by using the following formula:


(1) $$\begin{array}{r} \text{Rad}-\text{score}=\;c_{1}F_{1}+c_{2}F_{2}+\ldots +c_{i}F_{i} \end{array}$$

Where *F*_*i*_ is the selected radiomics feature, *c*_*i*_ is the LASSO coefficient of *F*_*i*_. Then, the assessment method of the logistic regression model is the receiver operating characteristic (ROC) curve and its area under the curve (AUC).

### Prognostic Validation of Radiomics Signature

We calculated Rad-score for each patient with ESCC and grouped them according to two rules. (1) The patients were divided into high-risk and low-risk groups based on the median Rad-score. (2) Patients with median scores were placed in high-risk groups. The radiomics signature discriminative performance of the survival status was assessed according to the overall distribution of ESCC patients. And then, the potential association of radiomics signature and clinical feature with PFS was assessed in the training cohort and validated in the validation cohort. Kaplan–Meier survival analysis was used in these two cohorts. Stratified analyses were implemented to determine the PFS in high-risk and low-risk patient subgroups. Univariate Cox Proportional Hazards Models were performed to explore the C-index of the radiomics signature.

### Performance of TNM Staging and Clinical Nomograms in the Training Cohort Before and After Addition of Rad-Score

The nomogram with the predicting model was based on the multivariable logistic regression analysis. The following candidate factors: TNM stage (dummy variable: “0” for I, “1” for II, “2” for III), the status of clinical features, and Rad-scores were involved in a diagnostic model for preoperative prediction of ESCC. The nomogram is a graphical representation of this prediction model in the training cohort. The prognostic performance of TNM staging and clinical nomograms in the training cohort before and after the addition of the Rad-score was quantitatively measured using Harrell's concordance index (C-Index), which is commonly used for the evaluation of the discriminative power of prognostic models (Harrell, 2018). The value of the C-index could range from 0.5, which indicated no discriminative ability, to 1.0, which showed perfect ability to distinguish between the patients who suffered disease progression or death and those who did not. Bootstrap analyses with 1,000 resample were applied to obtain a C-index with a 95% confidence interval (CI) (Canty and Ripley, 2021) corrected for potential overfitting. The calibration curves were drawn for assessing the agreement between the predicted probability of 3-year PFS and actual 3-year PFS (Pencina et al., 2011).

### Nomogram Validation in the Validation Cohort

The prognostic performance of TNM staging and clinical nomograms in the validation cohort before and after the addition of the Rad-score was tested by the above method. The calibration curve and C-index were calculated through multivariable Cox proportional hazard regression analyses. The decision curve analysis (DCA) was introduced to evaluate the quantified net benefit of our prediction model in the validation cohort (Vickers et al., 2008; Shen et al., 2018).

### Association of Radiomics Features With Clinical Data

A heat map analysis was used to evaluate the associations between clinical data and radiomics features.

### Statistical Analysis

All the statistical analyses were performed using IBM SPSS software (version 26; IBM Corp, Armonk, NY, USA), Matlab 2016b (Mathworks, Natick, USA), and R software (version 4.1.1, Boston, MA, USA). In this study, 2-sample *t*-test was applied to confirm whether differences between death and censoring groups in the primary cohort. The differences in gender, age, TNM stage, smoking status, drinking status, location, genetic history, invasion degree and metastasis between the training and validation data sets were assessed by using the χ^2^-test. The following R packages were used: the glmnet package was used for the LASSO logistic regression model analysis, the pROC package was used for the ROC curves, the Hmisc package was used for the comparisons between the C-indices, the survival package was used for Kaplan–Meier survival analyses, the rms package was used for the nomograms and calibration curves, the rmda package was used to implement the DCA, and the gplots and pheatmap packages were used for heat maps.

## Results

### Clinical Characteristics of All the Patients

A total of 272 consecutive patients who met the criteria (192 men and 80 women; mean age, 60.27 years ± 7.43) were included and divided into two cohorts by a ratio of 3:1 using computer-generated random numbers. A total of 204 patients were enrolled in the training cohort (146 men and 58 women; mean age, 60.22 years ± 7.28), while 68 patients were enrolled in the independent validation cohort (46 men and 22 women; mean age, 60.44 years ± 7.90). The clinical characteristics with statistics of the training and validation cohorts are summarized in Table 1. No significant differences were found between these two cohorts in terms of gender, age, history of smoking and drinking, location, genetic history, invasion degree, lymph node metastasis, and overall TNM Stage (*p* = 0.255–0.887). The median PFS was 35.35 months (range, 2–75 months).

### Radiomics Feature Selection and Radiomics Signature Building

**Figure 1:**
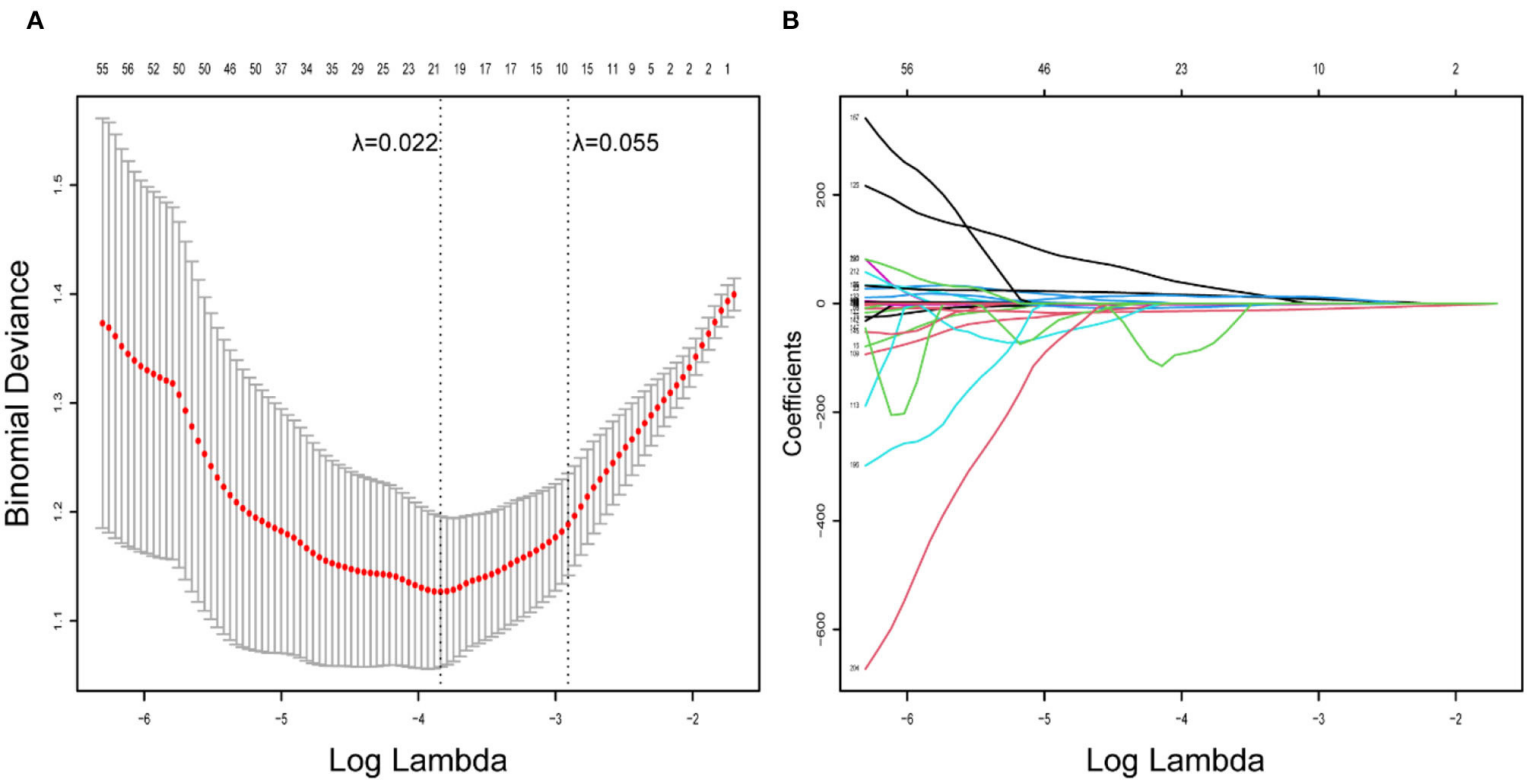
Radiomics feature selection using LASSO logistic regression model. **(A)** Identification of the optimal penalization coefficient lambda (λ) in the LASSO model used 10-fold cross-validation and the minimum criterion. As a result, a λ value of 0.022 was selected. **(B)** LASSO coefficient profiles of the 221 radiomics features.

**Figure 2:**
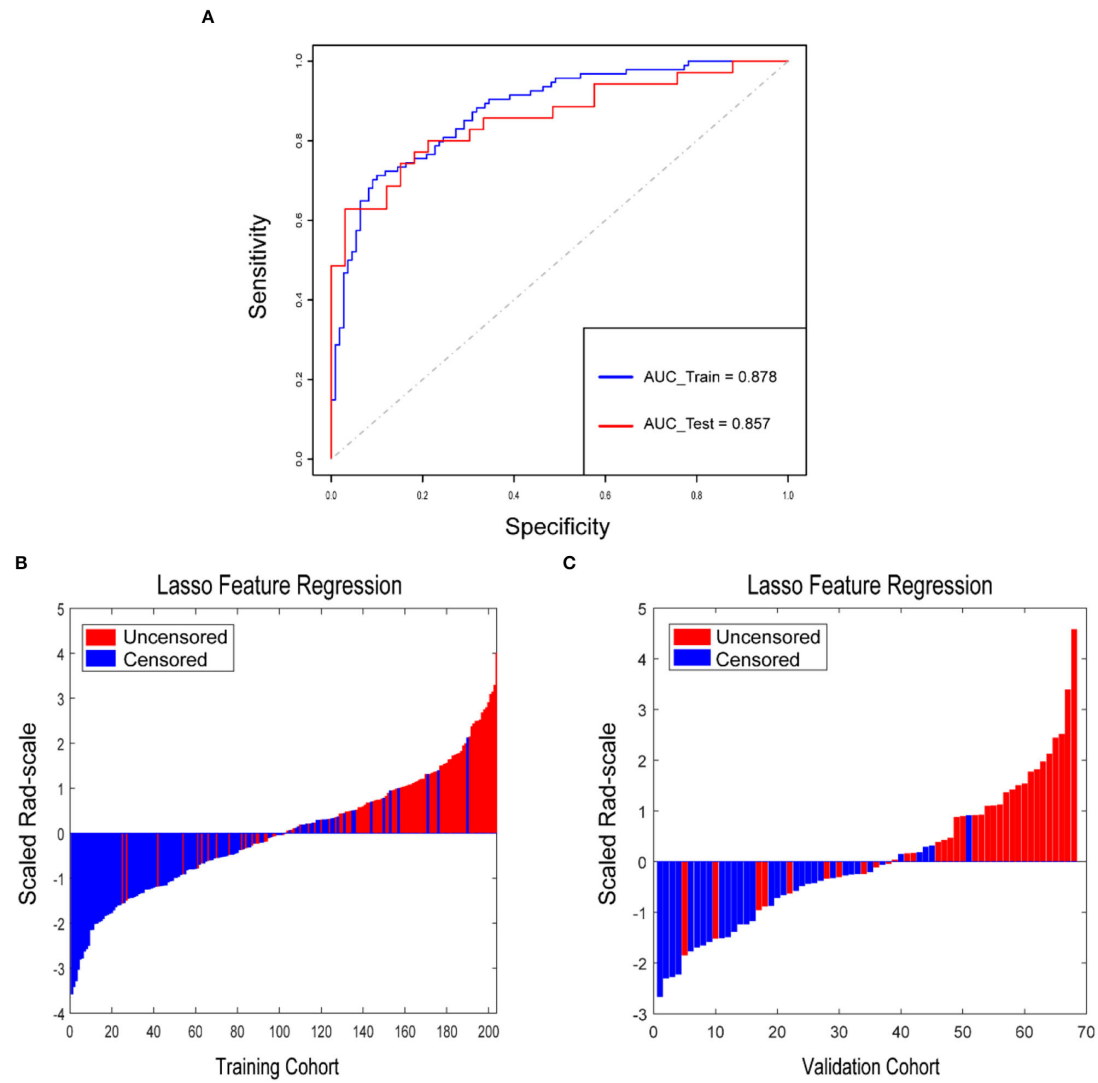
Rad-score for each patient in the training cohort and validation cohort. **(A)** ROCs were employed to assess the radiomics signature discriminative performance of the survival status. ROC in the training cohort with 0.878 (95% CI: 0.831–0.924, sensitivity = 71.3%, specificity = 90.0%); ROC in the validation cohort with 0.857 (95% CI: 0.767–0.947, sensitivity = 62.9%, specificity = 97.0%). Rad-score for each patient in the training cohort **(B)** and validation cohort **(C)**. Blue bars show scores for patients who survived without disease progression or were censored, while red bars show scores for those who experienced progression or died.

A total of 954 features were extracted from CT images and might contain many redundant and highly correlated features. To find out robust and valuable features, we performed the following steps: First, 221 features were selected by univariate statistical tests (*p* < 0.05) (Table 2). Then, based on the LASSO logistic regression algorithm approach in the training cohort, we selected the features with non-zero coefficients. As a result, 21 radiomics features were screened out from 221 features (Table 3). The procedures of parameter tuning and feature space reduction of the regression model are illustrated in Figure 1. Then the 21 features were selected to build the radiomics signature and involved in the Rad-score-based prognostic model. The discriminative ability of the survival status based on radiomics signatures was assessed by ROC in both cohorts, respectively (Figure 2A).

**Table 2:** Radiomics features selection results based on the ANOVA.

**Result category**	**CT**
Number of selected features	221
The best-performance feature	HLL-original_glcm_InverseVariance
	(*p* = 2.316589e-04)

**Table 3:** Radiomics signature selection results with descriptions.

**Feature name**	**Feature coefficient**
HHL_firstorder_Skewness	0.066
HLH_firstorder_Median	−1.812
HLH_glszm_SmallAreaEmphasis	−13.697
HLH_glszm_ZoneEntropy	0.092
HLL_glcm_ClusterShade	−0.004
HLL_glcm_InverseVariance	−6.470
HLL_glszm_GrayLevelNonUniformityNormalized	−0.612
HLL_glszm_SizeZoneNonUniformityNormalized	15.084
HLL_gldm_SmallDependenceHighGrayLevelEmphasis	−0.0008
HLL_ngtdm_Complexity	−0.0007
LHH_glszm_LargeAreaLowGrayLevelEmphasis	1.02e-06
LHH_gldm_DependenceNonUniformityNormalized	31.635
LHH_ngtdm_Busyness	0.001
LHL_glcm_Idn	12.445
LHL_glszm_LargeAreaHighGrayLevelEmphasis	−1.42e-10
LHL_gldm_SmallDependenceLowGrayLevelEmphasis	−82.462
LLH_firstorder_Energy	9.77e-10
LLH_glcm_Contrast	0.029
LLH_glszm_SizeZoneNonUniformity	1.37e-05
LLH_ngtdm_Complexity	5.66e-05
LLL_gldm_LargeDependenceHighGrayLevelEmphasis	2.44e-06

*Skewness:The asymmetric distribution of the Mean value. Depending on where the tail is elongated and the mass of the distribution is concentrated, it can be positive or negative*.

*Median:The median gray level intensity within ROI*.

*Small Area Emphasis (SAE): A measure of the distribution of small size zones, with a greater value indicative of more smaller size zones and more fine textures*.

*Zone Entropy (ZE): The degree of instability and variation in spatial and regional differences of the image distribution range*.

*Cluster Shade: A measure of skewness and uniformity of the GLCM. A higher cluster shade implies greater asymmetry about the mean*.

*Size Zone Non-Uniformity Normalized: The variability of size zone volumes throughout images, with a lower value indicating more homogeneity among zone size volumes in images. it's the normalized version of the SZN formula*.

*Small Dependence High Gray Level Emphasis (SDHGLE): Measures the joint distribution of small dependence with higher gray-level values*.

*Large Area Low Gray Level Emphasis (LALGLE): The proportion in images of the joint distribution of larger size zones with lower gray-level values*.

*Dependence Non-Uniformity Normalized (DNN): Measures the similarity of dependence throughout images, with a lower value indicating more homogeneity among dependencies in images. This is the normalized version of the DLN formula*.

*Busyness: A measure of the change from a pixel to its neighbor. A high value for busyness indicates a ‘busy’ image, with rapid changes of intensity between pixels and their neighborhood*.

*IDN (inverse difference normalized): Another measure of a local homogeneity of images. Unlike Homogeneity1, IDN normalizes the difference between neighboring intensity values by dividing over the total number of discrete intensity values*.

*Large Area High Gray Level Emphasis (LAHGLE): The proportion in images of the joint distribution of larger size zones with higher gray-level values*.

*Small Dependence Low Gray Level Emphasis (SDLGLE): Measures the joint distribution of small dependence with lower gray-level values*.

*Energy: The sum of squares of gray level intensity within ROI*.

*Contrast: A measure of local intensity variation, favoring values away from the diagonal (i=j). A larger value correlates with a greater disparity in intensity values among neighboring voxels*.

*Large Dependence High Gray Level Emphasis (LDHGLE): Measures the joint distribution of large dependence with higher gray-level values*.

### Prognostic Validation of Radiomics Signature

**Figure 3:**
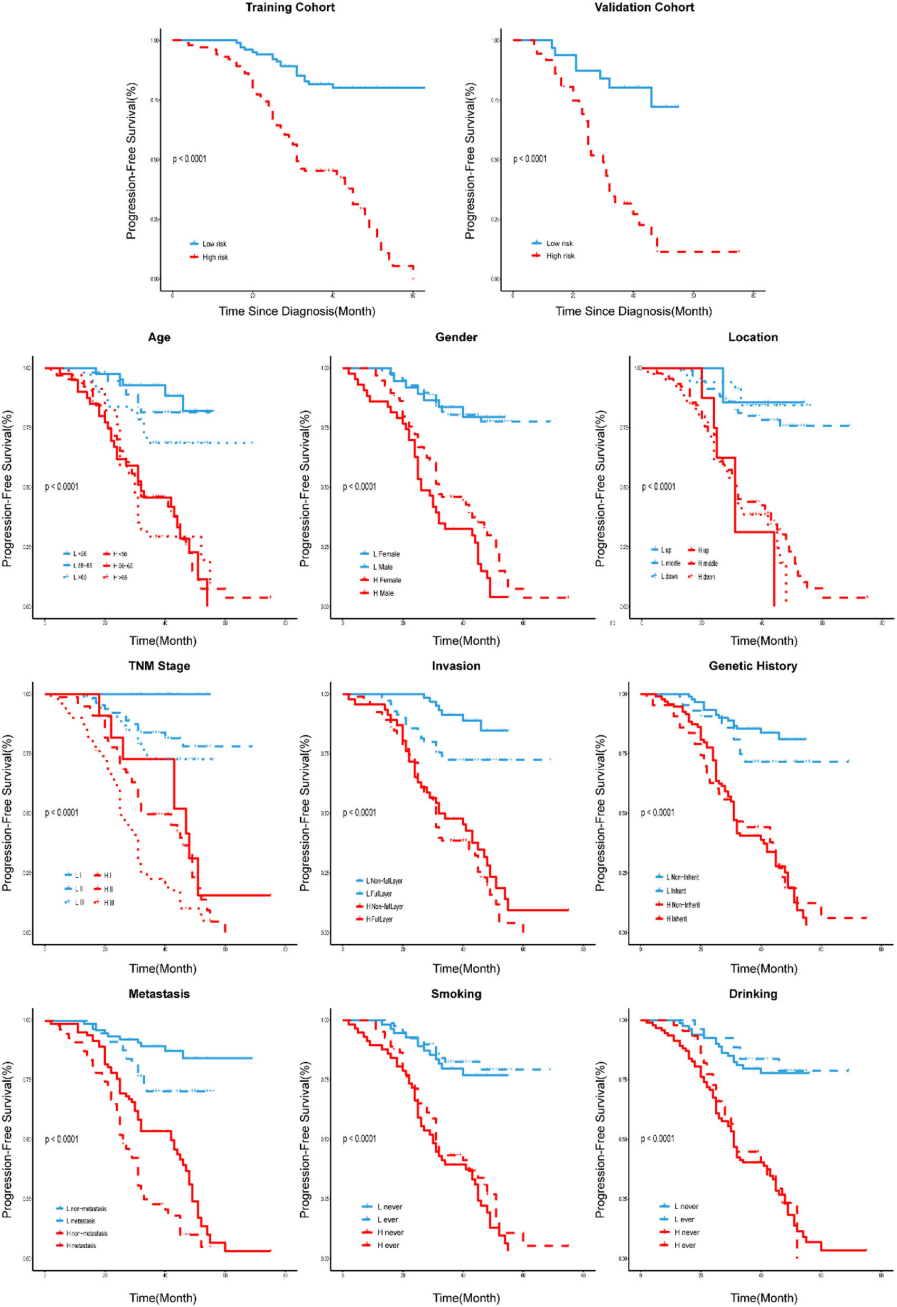
Stratified analyses were performed to estimate PFS in various subgroups, comparing high-risk patients and low-risk patients.

Rad-score for each patient in the training cohort and the validation cohort correspondingly showed that the higher the Rad-score, the greater the probability of death (Figures 2B,C). Besides, in the training cohort, the radiomics signature from CT images yielded the highest C-index, which was 0.758 (95% CI: 0.708–0.808). In the validation cohort, the radiomics signature from CT images yielded a C-index of 0.748 (95% CI: 0.656–0.840). It showed a significant discrimination between the PFS of high-risk and low-risk patients (Figure 3).

### Performance of TNM Staging and Clinical Nomograms in the Training Cohort Before and After the Addition of Rad-Score

**Figure 4:**
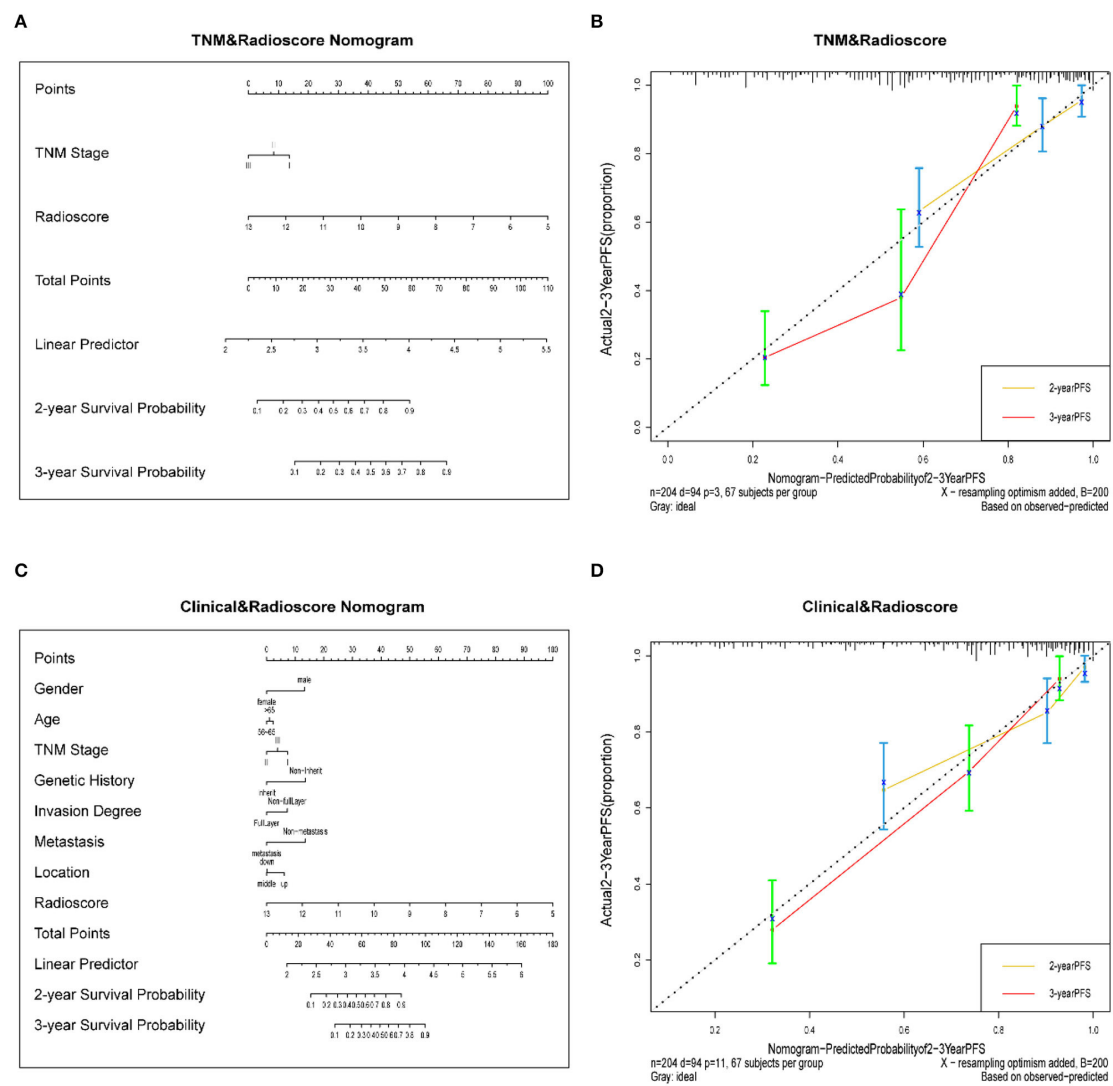
**(A)** A radiomics nomogram integrated the radiomics signature from CT images with the TNM staging system in the training cohort. **(B)** Calibration curve of the radiomics nomogram. The diagonal dotted line represents an ideal evaluation, while the yellow and red solid lines represent the performance of the nomogram. Closer fit to the diagonal dotted line indicates a better evaluation. **(C)** Adding Age, gender, invasion degree, location, genetic history, and metastasis to the radiomics nomogram. **(D)** Calibration curve of the radiomics nomogram with the addition of Age, gender, invasion degree, location, genetic history, metastasis.

We developed a radiomics nomogram that integrated the radiomics signature from the CT images with the traditional TNM staging system, which yielded a C-index of 0.603 (95% CI: 0.549–0.657). This nomogram significantly improved the discrimination ability in evaluating PFS (C-index: 0.770; 95% CI: 0.721–0.819) than TNM staging system (*p* < 0.05; Figure 4A), and showed good calibration as well (Figure 4B). Moreover, a radiomics nomogram was created by integrating the radiomics signature from the CT images with all clinical data, whose nomogram yielded a C-index of 0.680 (95% CI: 0.626–0.734). We found that the radiomics nomogram possessed good calibration and seemed to be more accurate than the clinical nomogram for evaluating PFS (C-index: 0.792; 95% CI: 0.748–0.836) with a *p* < 0.05 (Figures 4C,D).

### The Validation of Nomograms in the Validation Cohort

**Figure 5:**
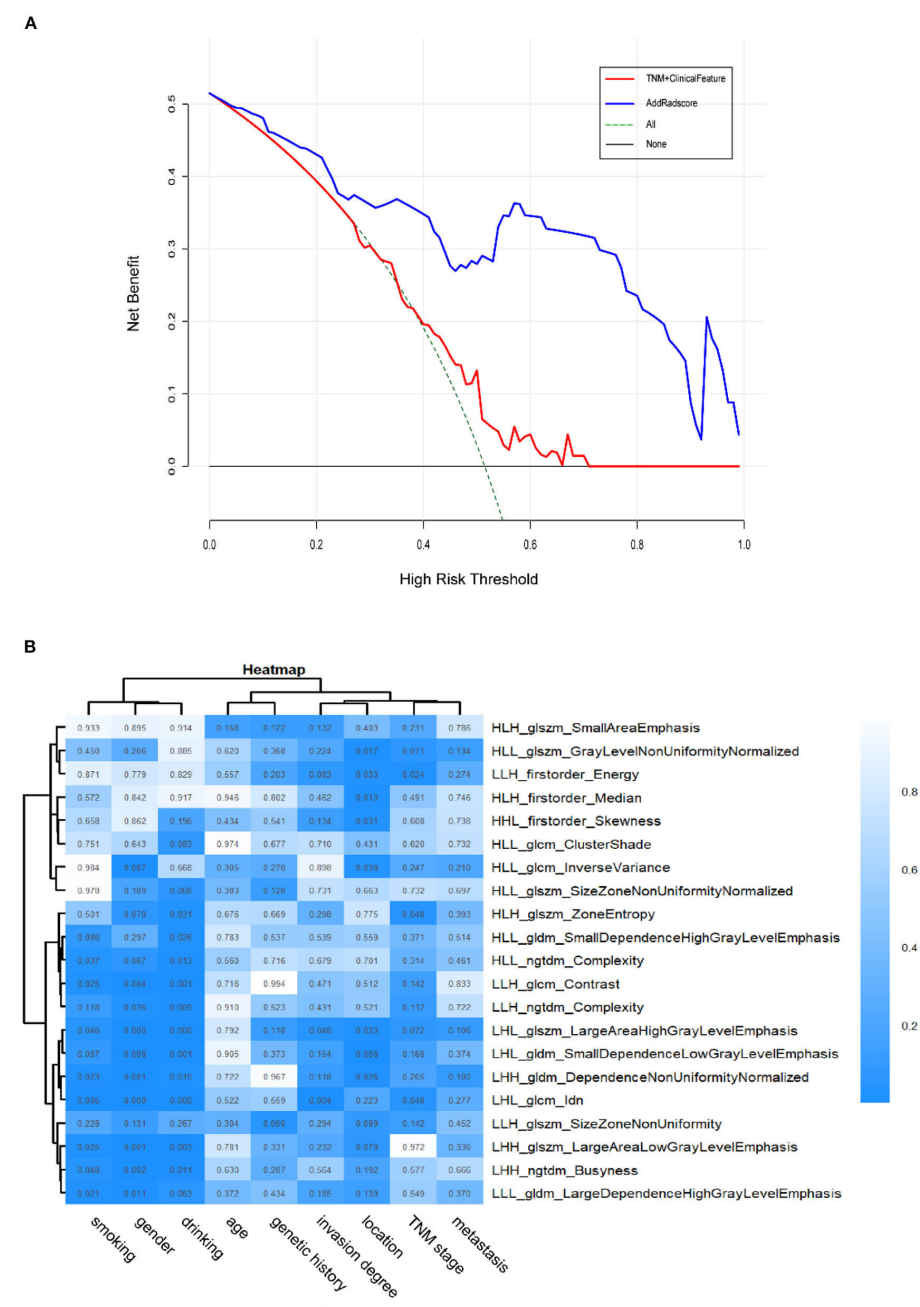
**(A)** The DCA of the radiomics-comparison-based nomogram. The black dotted line describes the scheme of no treatment. The green dotted line describes the scheme of treatment. The red line represents our predictive model with only traditional TNM staging combined with clinical features. And the blue line represents our personalized prediction model that added Rad-score. The x-axis is the threshold probability and the y-axis is the net benefit. It can be seen the personalized prediction model with Rad-score added had a better net benefit than the traditional predictive model when the threshold is in the range of 0–0.9. Hence, the patient with ESCC would receive benefit from taking our CT-based radiomics nomogram guidance. **(B)** Heatmap of associations between selected radiomics features and clinical data. *p* < 0.05 indicates statistical associations, as determined using *t*-tests.

In the validation cohort, the C-index of the traditional TNM staging system is 0.572 (95% CI: 0.478–0.666). We integrated the radiomics signature with the TNM staging system to produce a radiomics nomogram, which showed an improvement over the TNM staging system alone (C-index: 0.760; 95% CI: 0.673–0.847). The calibration curve of probability in PFS evaluation showed good agreement between nomogram-evaluated and actual observation (Figure not shown). While the clinical nomogram yielded a C-index of 0.605 (95% CI: 0.501–0.709) in the validation cohort and was advanced by combining with radiomics signature (C-index: 0.779; 95% CI: 0.697–0.861). The calibration curves of this nomogram showed good agreement between nomogram-evaluated and actual survival (Figure not shown). The DCA for the prediction model derived from the addition of Rad-score before and after is presented in Figure 5A. It showed that the predictive model collaborated with Rad-score had a better net benefit than that with only traditional TNM staging combined with clinical features.

### Association of Radiomics Features With Clinical Data

The ESCC patients with similar patterns of radiomics expression were clustered through unsupervised clustering (Figure 5B). Then we organized a heat map to determine the association between radiomics features and clinical data (Figure 5B). The results showed significant correlations between signature features LHL_glcm_Idn, LHL_glszm_Large Area High Gray Level Emphasis with drinking (*p* < 0.001) as well as gender (*p* < 0.001). LHL_glcm_ldn was associated with smoking (*p* < 0.01). LHL_gldm_Small Dependence Low Gray Level Emphasis was associated with location (*p* < 0.01). LHL_glcm_Idn and LHL_glszm_Large Area High Gray Level Emphasis were associated with invasion degree (*p* < 0.05). HLH_glszm_Zone Entropy, LHL_glcm_Idn and LLH_firstorder_Energy were associated with overall stage (*p* < 0.05). In contrast, no radiomics feature was significantly associated with age, genetic history and metastasis (for all, *p* > 0.05). These *p*-values for all the correlations of radiomics features with clinical data are shown in Supplementary Table 1.

## Discussion

Here, we first developed and validated a new approach based on CT radiomics to evaluate PFS before treatment in ESCC (stage I-III). The radiomics signature from CT images demonstrated better prognostic performance than traditional clinical information alone. It could be competently differentiated between patients with high-risk and low-risk, who had significantly different 3-year PFS, and were defined according to the median Rad-score. The developed radiomics nomogram transcended the traditional TNM staging system and clinical nomogram alone.

In clinical practice, CT, magnetic resonance imaging (MRI), positron emission tomography (PET), and endoscopic ultrasound (EUS) have their own advantages and disadvantages in the staging of esophageal cancer or even cancer. But the use of these modalities is limited to their cost in both time and money. CT owns the highest cost performance of its high availability and non-invasive process. However, the traditional prognosis is dependent on the doctors' observation, which differs greatly according to the experience. Moreover, the evaluation from traditional clinical information is even more inadequate. It is believed that there is still a lot of digital data that can be deeply excavated through the radiomics methodology and used for judgment conversely. Therefore, we analyzed all acquired CT images and constructed a CT-based radiomics signature. And the results confirmed our expectations that the radiomics signatures have the potential for evaluating prognosis in ESCC.

To build the radiomics signature, we selected 21 potential predictors from 954 candidate features by selecting highly correlated features with event outcomes and LASSO logistic regression. The radiomics features obtained are generally accurate. Because the regression coefficients of most features have shrunk toward zero during model fitting. It allowed the identification of features that had the strongest association with PFS (Ndhlovu et al., 2013) and avoided overfitting (Hepp et al., 2016). The radiomics signatures could reveal adequate discrimination both in the training cohort (C-index, 0.758) and the validation cohort (C-index, 0.748). Additionally, the selected features were used to improve radiomics signature and Rad-scores. We sorted the Rad-scores of all the patients with the labeled living status in Figure 2B, suggesting that the Rad-score could potentially differentiate the two types of patients. Other related statistical analyses also supported that the radiomics signature could be used as a biomarker in the prognosis of ESCC. We found that compared to the traditional TNM staging system and clinical nomogram, the radiomics signature dominated our nomogram in the training and validation cohorts. It means the radiomics signature has better discrimination and prognosis than classical radiologists, indicating the clinical importance of our findings due to the traditional clinical information and TNM staging are routinely used in clinical practice (Li et al., 2015; Wu et al., 2015).

Generally, doctors use the traditional TNM staging system for risk prediction and treatment planning. However, there were apparent differences in PFS with the same clinical identified disease stage, indicating that tumor heterogeneity would affect the survival outcomes. Patients with ESCC (stage I-III) with shorter PFS may benefit from the prognostic model because they may give up aggressive treatments to avoid suffering and overspending. Here, we developed the radiomics features possessing better prognostic ability than the traditional TNM staging system for pretreatment of PFS in the validation and training cohorts. Our study focused on the patients with stage I-III tumors (Table 1), and the patients with stage I accounted for a small proportion (9.3% in the training cohort, 13.2% in the validation cohort). Consequently, it might be not easy to stratify PFS accurately since the similar information of clinical stage. Additionally, the traditional TNM stage mainly reflects cancer patients' clinicopathologic features, such as tumor size, lymph node involvement, and distant metastasis status. They do have prognostic value in tumor treatment but neglected the intratumor heterogeneity, which was deemed as a crucial factor for tumor progression and prognosis (Yan et al., 2019). As a result, it provided an inefficient nomogram performance in both the training cohort (C-index, 0.603) and the validation cohort (C-index, 0.572). While the radiomics approach extracted the features of the entire tumor from medical images, which produced a more comprehensive way to involve the intratumor heterogeneity non-invasively. It might be why the combination of radiomics signatures and traditional TNM staging could provide a better nomogram performance in both the training cohort (C-index, 0.770) and validation cohort (C-index, 0.760). Hence, the radiomics signatures could assist the prognosis for ESCC complementarily to the traditional TNM staging.

Previous studies reported that clinical information including gender, pathological type, tumor differentiation, depth of invasion, and regional lymph node metastasis was associated with overall survival (OS) outcomes through univariate analysis. While multivariate analysis showed that pathologic type, depth of invasion, and regional lymph node metastasis were the independent predictors of OS (Liu et al., 2019). Besides, the tumor volume of ESCC could be used as an important prognostic factor for radiotherapy and chemotherapy assessment (Chen et al., 2013; Li et al., 2013; Chen Y. et al., 2016). Therefore, we exploited a clinical nomogram that combined available risk factors (age, gender, invasion degree, location, genetic history, metastasis) with the overall stage, but it doesn't exhibit well (C-index of training cohort, 0.680; C-index of validation cohort, 0.605). Then, we developed the nomogram by combining the radiomics signature in both the training cohort (C-index, 0.792) and the validation cohort (C-index, 0.779). This process suggested that radiomics signatures have important prognostic value for patients with ESCC.

Unlike the traditional methods, radiomics system is a non-invasive and low-spending approach, which could provide new insights into the associations between intrinsic tumor properties and biological behaviors. We analyzed the relationship between radiomics features and tumor-associated characteristics and observed some radiomics features were related to the general information of patients (gender, drinking, or smoking information, Figure 5B). Additionally, our radiomics system showed some radiomics features associated with invasion degree as well (Figure 5B). As a result, the present study may provide some different insights into the mechanisms of lymphatic metastasis of ESCC, which require future investigation.

There were several limitations in our study. First, we used thick-slice CT images rather than thin-slice images to extract radiomics signatures. Zhao et al. (2016) found that thin-slice images could reflect texture features of tumors more complete than thick-slice images. For the measurement of tumor volumes, thin-slice images had less measurement variability. We will further study the effect of thin-slice CT images for the staging of ESCC and confirm whether the performance is comparable with thick-slice images. Second, all data involved in this study are derived from the same hospital, resulting in the lack of multi-center validation. Further investigations on the applicability to the patients of other institutions are still required. Third, the analysis did not cover two-way or higher-order interactions of the radiomics features. If the interaction(s) strongly associated with the outcomes were applied, the prognostic performance of our nomogram might be significantly improved. However, revealing the interactions of multiple factors is challenging. In brief, our study clearly showed that the radiomics approach is potential for the prognosis of ESCC patients.

## Data Availability Statement

The raw data supporting the conclusions of this article will be made available by the authors, without undue reservation.

## Ethics Statement

The studies involving human participants were reviewed and approved by the Shanxi Medical University Review Board. Written informed consent to participate in this study was provided by the patient/participants or patient/participants' legal guardian/next of kin.

## Author Contributions

TY conceived the study, designed the experiments, analyzed the data, and wrote the manuscript. BW and YC edited the manuscript. LL, MP, ZY, QW, and SZ supervised data analysis. XZ provided clinical information and coordinated and performed segmentation of CT images. LW, HL, and YM performed the statistical analyses. All authors accessed the study data and reviewed and approved the final manuscript.

## Funding

This work was supported by funding from the National Natural Science Foundation of China (62176177, 81702449); the Fundamental Research Program of Shanxi Province (20210302123292, 20210302123112), the Research Project Supported by Shanxi Scholarship Council of China (2021-039), the Central Guidance on Local Science and Technology Development Fund of Shanxi Province (YDZJSX2021A018), the Shenzhen Project of Science and Technology (JCYJ20190813094203600).

## Conflict of Interest

The authors declare that the research was conducted in the absence of any commercial or financial relationships that could be construed as a potential conflict of interest.

## Publisher's Note

All claims expressed in this article are solely those of the authors and do not necessarily represent those of their affiliated organizations, or those of the publisher, the editors and the reviewers. Any product that may be evaluated in this article, or claim that may be made by its manufacturer, is not guaranteed or endorsed by the publisher.
